# Super-resolution for *asymmetric resolution* of FIB-SEM 3D imaging using AI with deep learning

**DOI:** 10.1038/s41598-018-24330-1

**Published:** 2018-04-12

**Authors:** Katsumi Hagita, Takeshi Higuchi, Hiroshi Jinnai

**Affiliations:** 1 Department of Applied Physics, National Defense Academy, Yokosuka, 239-8686 Japan; 2 Institute of Multidisciplinary Research for Advanced Materials, Tohoku University, 2-1-1 Katahira, Aoba-ku, Sendai, 980-8577 Japan

## Abstract

Scanning electron microscopy equipped with a focused ion beam (FIB-SEM) is a promising three-dimensional (3D) imaging technique for nano- and meso-scale morphologies. In FIB-SEM, the specimen surface is stripped by an ion beam and imaged by an SEM installed orthogonally to the FIB. The lateral resolution is governed by the SEM, while the depth resolution, i.e., the FIB milling direction, is determined by the thickness of the stripped thin layer. In most cases, the lateral resolution is superior to the depth resolution; hence, *asymmetric resolution* is generated in the 3D image. Here, we propose a new approach based on an image-processing or deep-learning-based method for super-resolution of 3D images with such *asymmetric resolution*, so as to restore the depth resolution to achieve symmetric resolution. The deep-learning-based method learns from high-resolution sub-images obtained via SEM and recovers low-resolution sub-images parallel to the FIB milling direction. The 3D morphologies of polymeric nano-composites are used as test images, which are subjected to the deep-learning-based method as well as conventional methods. We find that the former yields superior restoration, particularly as the asymmetric resolution is increased. Our super-resolution approach for images having *asymmetric resolution* enables observation time reduction.

## Introduction

The increased speed of high-throughput meso-scale three-dimensional (3D) observations is of general interest for both material science and medical applications^1–3^. Similarly, utilization of artificial intelligence (AI) technologies in such research areas is attracting widespread interest^4–11^. For future industrial innovation, application of AI technologies such as deep learning (DL) in addition to high-throughput 3D observation and high-speed analysis is urgent.

Recently, DL-based AI technologies have experienced a sudden, large increase in popularity with regard to many business and industrial applications, in addition to academic research^4–11^. The high performance of DL for image recognition^12^ compared to conventional approaches^13^ is well known, and DL has become a core technology for self-driving cars^8^. “AlphaGo” is another famous DL application^9^. DL is a type of machine learning by neural networks^10,11^. Three major tasks of machine learning are solution of clustering (with unsupervised learning), classification (with supervised learning), and regression problems. Super-resolution (SR) is classified as a regression problem. From the perspective of information science, particularly computer vision, SR is an ill-posed inverse problem involving recovery of information lost by down-sampling^14^. For SR, a description of the relationship between low- (input) and high-resolution images (output) is required. Before DL, SR rules were developed by analyzing neighboring pixels and/or pairs of low- and high-resolution images. Very recently, many highly successful DL approaches to SR have been reported^15–18^, e.g., the Super-Resolution Convolutional Neural Network (SRCNN)^15^ and Generative Adversarial Network for SR (SRGAN)^16^. In DL-based methods, the relationship between the low-/high-resolution images is described by deep CNN or GAN. Dong *et al*. have theoretically explained why the SRCNN yields better performance than example-based SR methods, such as sparse-coding-based SR^19,20^, in terms of CNN^15^. Further, Ledig *et al*. have reported that SRGAN exhibits superior performance to SRCNN^16^ for standard benchmark image datasets such as “Set5”^21^, “Set14”^22^ and “BSD100”^23^. Those researchers confirmed that SRCNN and SRGAN exhibit superior performance to conventional interpolation methods such as nearest neighbor, bicubic, and bilinear. Compared to the nearest neighbor method, bicubic and bilinear seem to have comparable performance to SRCNN and SRGAN. As some implementations using a DL software library such as TensorFlow^24^ have already been released, we can quickly perform network learning from big data regarding pairs of low-/high-resolution images.

Scanning electron microscopy equipped with a focused ion beam (FIB-SEM) is one of the most powerful tools for observation of polymer 3D structures. Compared with electron tomography^25–31^, this technique has the advantage of observing a wide volume region. FIB-SEM has a lower resolution than electron tomography due to its large stripping pitch (several to 20 nm, in general) for soft materials. However, FIB-SEM is capable of obtaining much larger observation volume than electron tomography does. These two 3D imaging techniques are complimentary. We note that it was possible to considerably reduce the stripping pitch by staining the specimens so that the rubbery part became glassy in the present study. In this technique, a sample surface is stripped via FIB and the cross-sectional surface is observed via SEM at high resolution (of the order of a few nanometers). The FIB stripping process and 2D SEM observation are repeated multiple times; then, the SEM 2D images are stacked to generate a 3D volume image. In this study, the SEM image resolution is defined as the lateral resolution (*x*- and *y*-directions), while the direction perpendicular to the lateral plane is the depth direction (*z*-direction). Thus, the lateral resolution is determined using SEM, whereas the depth resolution corresponds to the thickness of the FIB-stripped surface. Soft materials are mostly non-conductive and easily thermally decomposed; therefore, the depth resolution (several to tens of nanometers) is often much lower than the resolution for the SEM observations (a few nanometers). Therefore, in most cases, the resulting 3D image exhibits *asymmetric resolution*. One of the most popular applications of FIB-SEM with regard to soft materials is imaging of filler-filled polymer nanocomposites (PNCs)^25–39^. In the case of silica nanoparticles (NPs) filling poly(styrene-*ran*-butadiene) rubber (SBR), the FIB milling interval range is 10 to 20 nm. Because the local morphology of the NP-filled SBR system can be considered isotropic, SR may be effective to recover local information. If so, it should constitute a very effective method for providing symmetric 3D resolution for FIB-SEM 3D data. In addition, SR can significantly reduce FIB-SEM measurement time by decimating some SEM cross-sectional images. This decimation can be achieved by increasing the thickness of the FIB stripped surface and, thus, reducing the number of SEM images required to generate the 3D data, the resolution asymmetry of which is then restored via SR.

**Figure 1:**
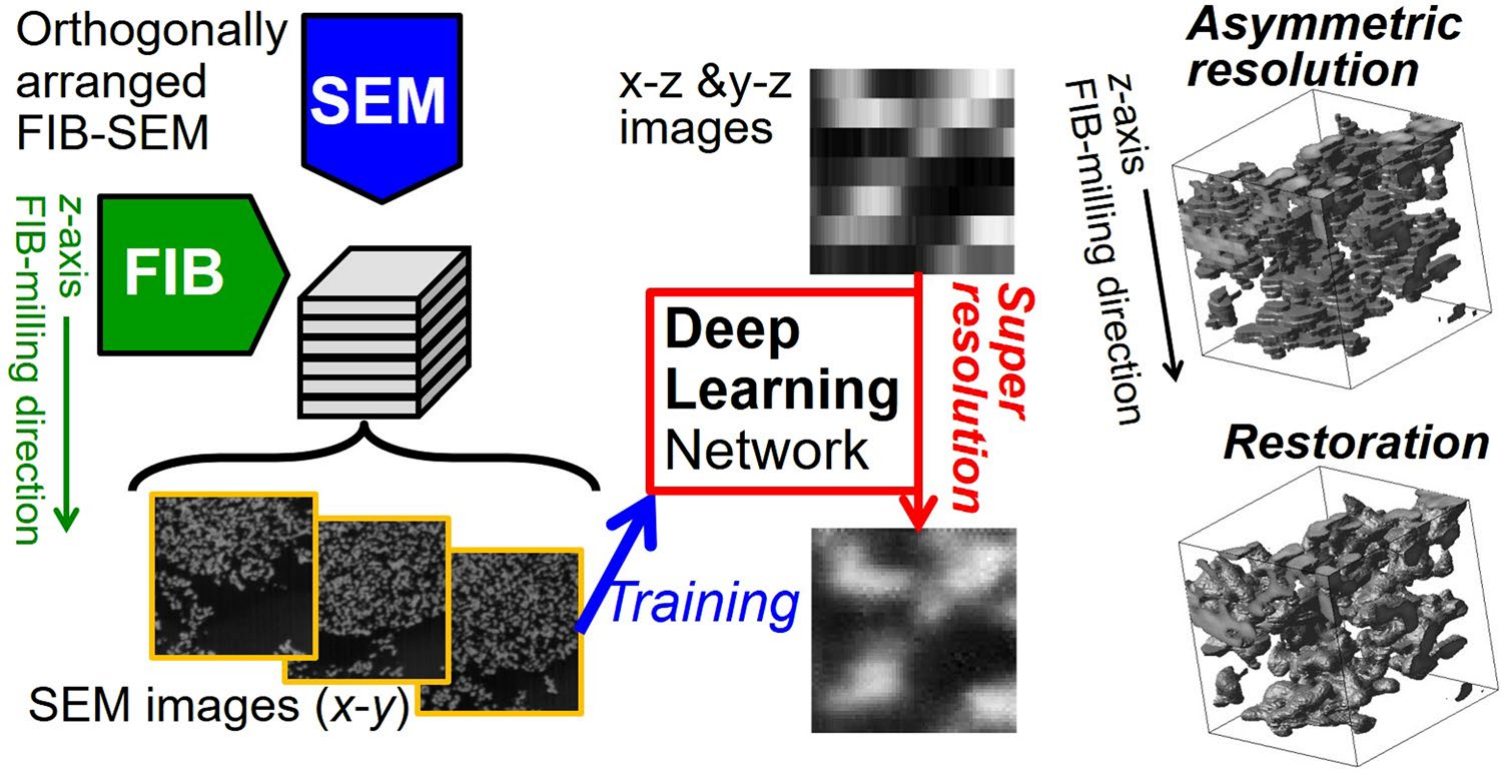
Illustration of our proposed SR approach for treatment of 3D image data with *asymmetric resolution*.

In this study, as a practical application to accelerate meso-scale 3D observations, we propose an SR technique for high-speed 3D observations with *asymmetric resolution*. An overview of this technique is given in Fig. 1. We believe that this method enables both acceleration and cost reduction of meso-scale 3D microscopy through use of an integrated system incorporating several 3D microscopy techniques. To demonstrate SR processing for meso-scale 3D observation, we perform FIB-SEM observation of filler-filled PNCs with *asymmetric resolution* in the *z*-direction. SR is then performed for 2D sub-images within the *x*-*z* and the *y*-*z* planes. A DL-based SR method is applied, with 2D sub-images within the *x*-*y* plane being used for the learning processes. The depth-to-lateral resolution ratio corresponds to the upscaling factor *n* of the SR processing. From the recovered sub-images in the *x*-*z* and the *y*-*z* planes, 3D volume data are reconstructed. Hence, we evaluate the dependence of the restoration of the image resolution on the *n* in the depth direction. In order to study the efficiency of the SR approach, we compare SR results obtained using conventional interpolation methods and DL-based methods. Further, we clarify the parameter settings, i.e., conditions, beneficial for each method.

From the viewpoint of rubber technologies, there is a high interest in filler morphologies; the FIB-SEM observation of filler-filled PNCs is a powerful tool, as well as small-angle X-ray scattering (SAXS) experiments^32–35^, to observe 3D volume data of NP morphologies. Although many studies have semi-quantitatively compared the behavior of SAXS data and 2D images of SEM and/or TEM micrographs, it is, in principle, impossible to calculate scattering functions from the 2D image. If 3D volume data are observed, the scattering functions can be approximately calculated. Then, we expect to use the SAXS data to improve the resolution of the 3D volume data. Namely, some modifications to the FIB-SEM 3D image are made so that the scattering function calculated from the (modified) 3D volume data agrees well with the actually measured SAXS data. Such a super resolution technique should shorten measurement time because one needs only a roughly stripping pitch for FIB-SEM observation.

The remainder of this paper is organized as follows. The results for the original and reconstructed 3D volume data and numerical evaluations of the various SR methods are presented in the next section (Results). A comparison of image-process-based and DL-based SR is presented. The final section (Methods) provides a brief description of the examined specimens, FIB-SEM observation methods, the SR procedures employed in this study, and the evaluation method.

## Results

**Figure 2:**
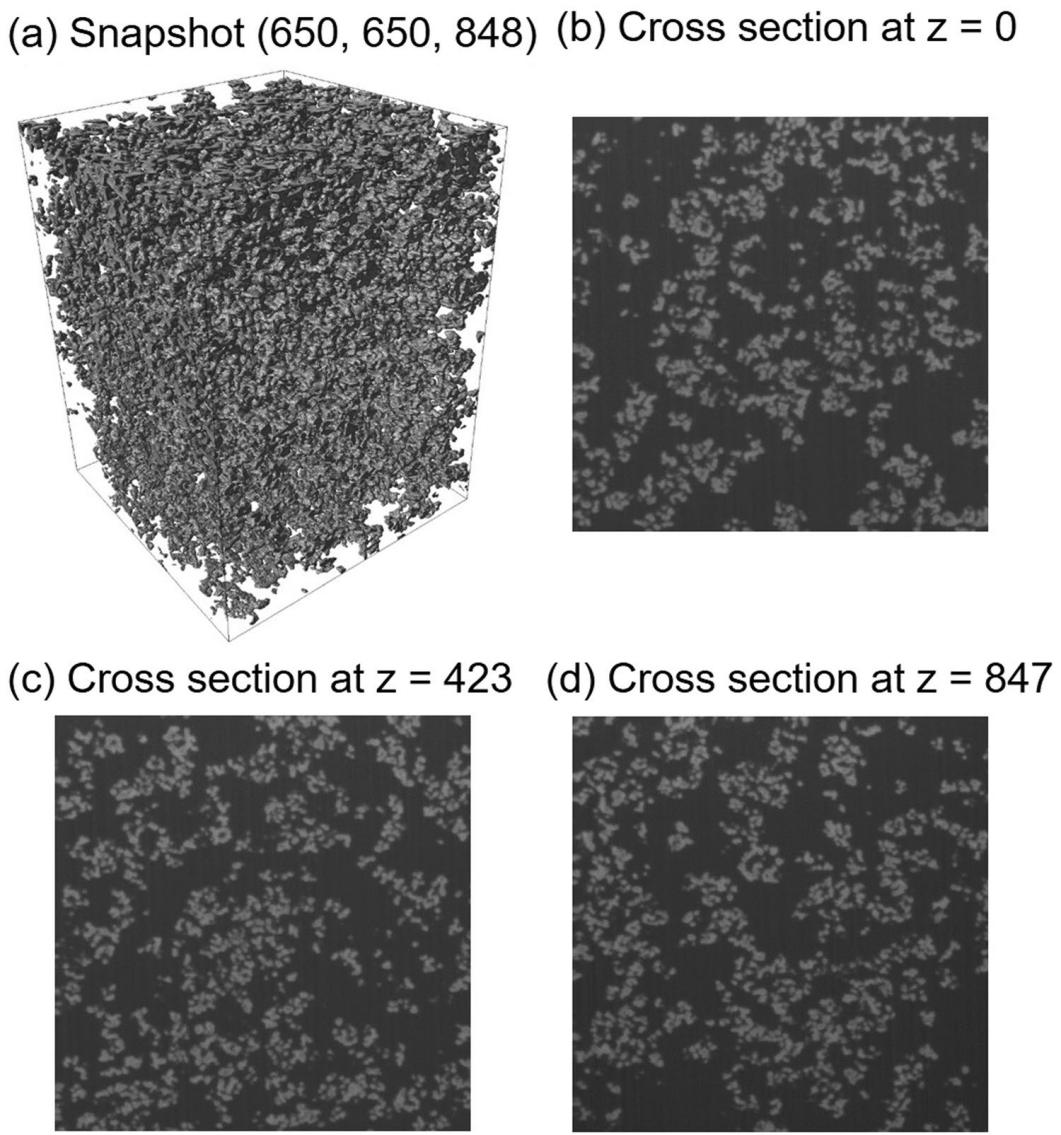
(**a**) Snapshot and (**b**−**d**) cross sections of 3D volume data used as reference. 2D images obtained via FIB-SEM are presented in (**b**−**d**).

We performed FIB-SEM observation of 3D volume data for NP-filled SBR (supplied by JSR Corporation). Note that the mechanical properties and small angle X-ray scattering curves of these specimens have been examined in detail in previous studies^32,35^. These volume data with 2-nm/pixel resolution were used as a reference for SR in the present study. Stripping with 2-nm pitch was achieved by converting the specimen into a glass state by the OsO_4_ staining of the rubbery matrix of filler-filled PNCs. In this case, local relaxations of NPs at the surface after milling were considered sufficiently small. A snapshot and some cross sections of a reference 3D image are presented in Fig. 2. In Fig. 2(a), we plotted the isosurface with a threshold value of 90 for the obtained 8-bit grayscale image. This threshold value was determined so as to reproduce the volume fraction of the NP-filled SBR. In Fig. 2(b–d), the original FIB-SEM images obtained at different depth positions are shown.

In this study, decimated volume images were generated by replacing *n* consecutive pixels with a representative value in the *z*-direction. The value $u(x,y,z)$ was obtained from $u(x,y,z)=u(x,y,\lfloor z/n\rfloor n+\lfloor n/2\rfloor )$, where $\lfloor X\rfloor$ is the floor function of *X*. In our test, cases with *n* values of 2, 4, and 8 were examined. We trained the SRGAN model for each upscaling factor (*n* *=* 2, 4, and 8) using the TensorFlow package^24^. Here, SRGAN learned from sub-images within the *x*-*y* plane. We then applied the trained SR to 2D sub-images within the *x*-*z* and the *y*-*z* planes. For comparison with conventional interpolation methods, we performed image-filter-based SR for 2D sub-images within the *x*-*z* and the *y*-*z* planes using the nearest neighbor, bicubic, and bilinear methods, along with the OpenCV 2.4 standard image processing library^40,41^.

**Figure 3:**
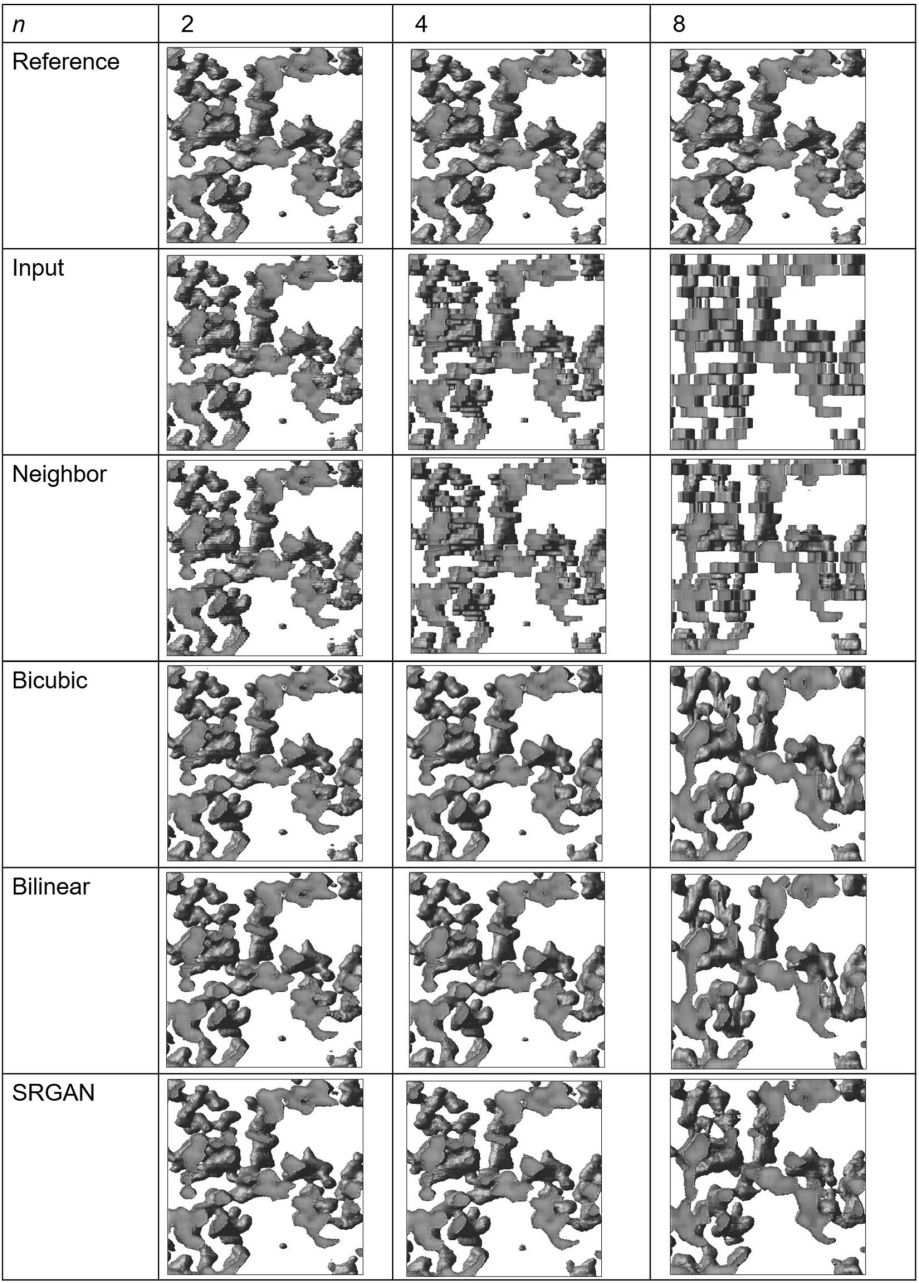
Ultra-thin sections in *x*-*z* planes for reference, input (low-resolution), and output images of our SR processing.

Figure 3 shows ultra-thin sections in the *x*-*z* planes for the reference, input (low-resolution images), and output images of our SR processing. The image size was (128, 128). Comparison of the three images with different *n* values for the input revealed that the difference in resolution is significant. In the case of *n* *=* 8, the SR results yielded by the bicubic and bilinear methods and SRGAN seemed to be improved.

Numerical evaluations based on root mean square (RMS) and peak signal-to-noise ratio (PSNR) calculations are presented in Tables 1 and 2. Table 1 lists the results obtained from 8-bit grayscale images with *n* *=* 2, 4, and 8. The bicubic and bilinear methods and SRGAN seemed to exhibit comparable performance. The same finding has been reported in several papers on DL-based SR^15–18^ for standard benchmark datasets^21–23^. However, for small *n* (= 2), the numerical values yielded by SRGAN were lower than those obtained through conventional methods. On the other hand, for large *n* (= 8), SRGAN yielded the best performance. Table 2 lists RMS and PSNR values obtained for 1-bit binary images, as a check of the binarization effect. The binary images were obtained with a threshold value of 90 for the 8-bit grayscale images. The order of the PSNR (and RMS) results exhibited similar trends for *n* *=* 2 and 4.

**Table 1:** Dependence of *n* on RMS (PSNR) for 8-bit grayscale image.

*n*	2	4	8
Input	0.0439 (39.0 dB)	0.0938 (32.4 dB)	0.172 (27.1 dB)
Nearest neighbor	0.0439 (39.0 dB)	0.0938 (32.3 dB)	0.211 (25.3 dB)
Bicubic	0.0252 (43.8 dB)	0.0415 (40.1 dB)	0.122 (30.0 dB)
Bilinear	0.0246 (44.0 dB)	0.0383 (39.4 dB)	0.121 (30.1 dB)
SRGAN	0.0320 (41.7 dB)	0.0421 (39.3 dB)	0.120 (30.2 dB)

**Table 2:** Dependence of *n* on RMS (PSNR) for 1-bit binary image with threshold value of 90 for 8-bit grayscale image.

*n*	2	4	8
Input	0.00191 (18.1 dB)	0.00314 (13.8 dB)	0.00453 (10.6 dB)
Nearest neighbor	0.00191 (18.1 dB)	0.00314 (13.8 dB)	0.00530 (9.18 dB)
Bicubic	0.00126 (21.7 dB)	0.00180 (18.6 dB)	0.00389 (11.9 dB)
Bilinear	0.00126 (21.7 dB)	0.00191 (18.1 dB)	0.00392 (11.8 dB)
SRGAN	0.00158 (19.7 dB)	0.00203 (17.6 dB)	0.00383 (12.0 dB)

Note that, in the case of *n* *=* 8, SRGAN yielded the best results for the 8-bit grayscale images, followed by the bilinear and bicubic methods. However, for the 1-bit binary images, although SRGAN again exhibited the best performance, the order after SRGAN was inverted, i.e., the bicubic method was superior to the bilinear technique. Hence, it can be concluded that the DL-based method is preferable for larger *n*. On the other hand, for small *n*, conventional image processing seems useful. For practical engineering, optimization of the balance between the observation and computing times for the SR process is important.

### Summary

A combination of FIB-SEM observation with *asymmetric resolution* and SR was proposed to achieve high-throughput 3D observation of PNCs. We expected that large FIB milling intervals would facilitate acceleration of the FIB-SEM observation, with the aid of SR processing to recover the gaps between the resolution in the FIB milling direction (depth resolution) and that in the SEM observation (lateral resolution). In this paper, we examined the dependence of the restoration performance on the upscaling factor (*n* *=* 2, 4, and 8) and on the SR method. Image-processing and the DL-based SR methods were considered. To obtain real, representative FIB-SEM 3D images, FIB-SEM observations of NP-filled SBR with 2-nm/pixel resolution were performed. As an implementation of a DL-based SR process, SRGAN^16^ with the TensorFlow^24^ library was used. SRGAN learnt from sub-images of SEM observations in the lateral direction and applied the SR process to sub-images parallel to the FIB milling direction, i.e., in the depth direction. After the SR process, a 3D volume image was reconstructed from the recovered sub-images. In addition, conventional image processing instead of SRGAN was applied to sub-images in the depth direction. We evaluated the RMS and PSNR as indexes of the SR performance. Hence, it was confirmed that use of an SR process yielded improved 3D volume images. In addition, we confirmed that SRGAN is superior to conventional image processing methods such as the bicubic and bilinear techniques for larger *n* (= 8). However, the improvements due to SR for the bicubic and bilinear methods, and SRGAN, were comparable when *n* was small. The SR approach for observation with *asymmetric resolution* can be applied to not only PNCs, but also phase-separated polymer systems of block copolymers. Moreover, reduction of observation time is beneficial for a broad range of research fields and industrial applications, including biomedical and mechanical engineering. When engineering FIB-SEM observation, establishing a balance between the observation and computing times for the SR process is important. Detailed studies and engineering optimization of selected parameters such as the sub-image sizes are in progress. Moreover, we are considering exploration of different network structures in order to achieve better performance and speed in our future research.

## Methods

As specimens for FIB-SEM observation, we used NP-filled SBR without end-functionalization, supplied by JSR Corporation. Details of the SBR used in this study are given in refs^32,35^. The average NP diameter was 18.8 nm and the NP volume fraction in the SBR was 16%. Analytical data on the SBR are given in ref.^32^. The total molecular weight, vinyl content of the butadiene part, and styrene unit content were, respectively, 189 kg/mol, 55%, and 20%. The glass transition temperature *T*_*g*_ was −36 °C. The NP-filled SBR compound had the following components: 100 per hundred parts of rubber (phr) SBR, 50-phr silica NP (Nipsil AQ, TOSOH SILICA), 4-phr silane coupling agent (Si75, EVONIK), 10-phr oil, 2-phr stearic acid, 1-phr *N*-(1.3-Dimethylbutyle)-*N*’-phenyl-p-phenylendiaminea, 1.5-phr diphenylguanidine, 1.8-phr *N*-Cyclohexyl-2-benzothiazylsufenamide, and 1.5-phr sulfur. After mixing in a 75-ml Plastomill (Toyo Seiki Seisakusho, Ltd.), this compound was cured at 160 °C for 40 min under press forming. Note that details of the dynamic viscoelastic properties, transmission electron microscopy images, and the ultra-small angle X-ray scattering spectrum are given in ref.^32^.

For FIB-SEM observation, the silica-NP-filled SBR was stained with osmium tetroxide (OsO_4_) crystalline for 1 day. Because OsO_4_ reacts with double bonds in the SBR and cross-links the SBR^42,43^, the stained SBR has sufficient tolerance for Ga ion and electron beam irradiation. In particular, the addition of Os into the SBR improves the SBR electron conductivity, effectively preventing sample charging due to the Ga ion and electron beam irradiation. In this study, the staining facilitated stripping of the SBR at several-nanometer pitches with the Ga ion beam. We believe that our super resolution method is applicable to wide variety of composite materials that are in a glassy state at room temperature. Alternatively, it may be also possible to perform FIB-SEM measurements at the cryogenic temperatures that convert soft materials, such as rubbery materials, into a glassy state. As shown in the present work, staining is also an excellent way to make the rubbery materials glassy.

We used an FIB-SEM (SMF-1000, SII Nano Technology, Inc., Japan) installed at the National Institute for Material Science, Japan. This instrument has both FIB and SEM installed orthogonally, so that the SEM view is from directly above the specimen. The FIB and SEM were operated at 30 and 0.5 kV, respectively. We obtained 880 images with (1000, 1000) pixels for each specimen through a repeated process of FIB sectioning and SEM observation at room temperature. The time required to measure one specimen was approximately 6 h. As the average FIB milling length was approximately 2 nm, the SEM magnification was chosen to yield a resolution of 2 nm/pixel. Because of the OsO_4_ staining, the FIB and SEM resolutions were symmetric in this particular case. Using ImageJ^44^, we performed image registration based on correlations of neighboring SEM images along the depth direction^45^. Consequently, we obtained volume data with (650, 650, 848) pixels.

In our SR procedure, the size of a unit sub-image was (56, 56) pixels. From a single SEM image with (1000, 1000) pixels, 289 (= 17 × 17) sub-images were obtained. Thus, the number *N*_L_ of sub-images for the learning process became 289 × 880/*n*. For *n* *=* 2, 4, and 8, *N*_L_ = 127160, 63580, and 31790, respectively. In training, low-resolution sub-images were generated by decimation from high-resolution sub-images through a down-sampling operation with a factor of 1/*n* in a certain direction (in our case, the *z*-direction). The low-resolution sub-images were (56, 56/*n*) pixels in size. For the SR processes, a single image with (650, 848) pixels in the *x*-*z* or *y*-*z* plane was divided into 276 (= 12 × 23) sub-images with (56, 56) pixels. In order to eliminate the difference between the edges of adjacent images, the edges of those images were overlapped. In the present study, the numbers of overlapped pixels between neighboring sub-images were 1 and 10 pixels in the *x*- or *y*-direction, and in the z-direction, respectively. We chose 10 pixels in the *z*-direction, because 10 is the smallest even integer larger than the maximum of the examined values of *n*. We applied SR processing for 179400 (= 650 × 276) sub-images within the *x*-*z* plane and 179400 sub-images within the *y*-*z* plane. 3D volume data were reconstructed from these 358800 sub-images.

For the DL-based SR, we used the SRGAN model. In practice, DL networks were implemented using the TensorFlow package (version 0.12.0)^24^. SRGAN learned from the large *N*_L_ within the *x*-*y* plane for each *n* (= 2, 4, and 8). On a machine employing one NVIDIA GeForce GTX 1080 graphics card, training with 500 epochs for *n* *=* 2, 4, and 8 required roughly 70, 35, and 18 h, respectively. In all cases, the cost function during training seemed to decrease before 50 epochs and saturate and fluctuate after 50 epochs. Consequently, we expended 500 epochs, which is ten times longer than 50 epochs.

For quantitative evaluation, we used the following root mean square (RMS) calculation of the difference between values for each pixel

1 $$RMS=\sqrt{\frac{\sum _{x,y,z}{(u(x,y,z)-{u}_{org}(x,y,z))}^{2}}{\sum _{x,y,z}u(x,y,z)}},$$

where *u*(*x*, *y*, *z*) denotes the value of the pixel at (*x*, *y*, *z*) and *u*_org_(*x*, *y*, *z*) is a reference data value. Further, the peak signal-to-noise ratio (PSNR) was used as a standard index to evaluate the goodness of the SR algorithms^15–23^. The PSNR (in dB) is defined as

2 $$PSNR=10\,{\mathrm{log}}_{10}\frac{MA{X}_{I}^{2}}{RMS},$$

where *MAX*_*I*_ is the maximum possible pixel value of the image. For 8-bit images and binary images, *MAX*_*I*_ is 255 and 1, respectively. In the present study, the significant digits of *RMS* and *PSNR* in Tables 1 and 2 were evaluated from the value of the area obtained by equally dividing the volume into eight equal parts.

### Data availability

The datasets generated during and/or analyzed during the current study are available from the corresponding author on reasonable request.

## References

[1] Occhetta P (2015). High-Throughput Microfluidic Platform for 3D Cultures of Mesenchymal Stem Cells, Towards Engineering Developmental Processes. Sci. Rep..

[2] Hongisto V (2013). High-Throughput 3D Screening Reveals Differences in Drug Sensitivities between Culture Models of JIMT1 Breast Cancer Cells. PLoS One.

[3] Bosch C (2015). FIB/SEM technology and high-throughput 3D reconstruction of dendritic spines and synapses in GFP-labeled adult-generated neurons. Front. Neuroanat..

[4] LeCun Y, Bengio Y, Hinton G (2015). Deep learning. Nature.

[5] Schmidhuber J (2015). Deep Learning in Neural Networks: An Overview. Neural Netw..

[6] Hinton GE, Salakhutdinov R (2006). Reducing the dimensionality of data with neural networks. Science.

[7] Russakovsky O (2015). Imagenet large scale visual recognition challenge. Int. J. Comput. Vis..

[8] Bojarski M (2016). End to End Learning for Self-Driving Cars. arXiv.

[9] Silver, D. *et al*. Mastering the game of Go with deep neural networks and tree search. *Nature* **529**, 484–489 (2016). 10.1038/nature16961 26819042

[10] Goodfellow, I., Bengio, Y. & Courville, A. Deep Learning. *MIT**Press*; http://www.deeplearningbook.org (2016).

[11] Géron, A. Hands-On Machine Learning with Scikit-Learn and TensorFlow. (O’Reilly Media 2017).

[12] Deng, J. *et al*. ImageNet: A large-scale hierarchical image database. *Proc*. *IEEE**Conference**on**Computer**Vision**and**Pattern**Recognition* 248–255 (2009).

[13] Krizhevsky A, Sutskever I, Hinton GE (2012). ImageNet Classification with Deep ConvolutionalNeural Networks. Advances in Neural Information Processing Systems.

[14] Yang, C.-Y., Ma, C. & Yang, M.-H. Single-image super-resolution: A benchmark. *European**Conference**on**Computer**Vision**(ECCV)* 372–386 (Springer, 2014).

[15] Dong C, Loy CC, He K, Tang X (2014). Image super-resolution using deep convolutional networks. IEEE Transactions on Pattern Analysis and Machine Intelligence (TPAMI).

[16] Ledig, C. *et al*. Photo-Realistic Single Image Super-Resolution Using a Generative AdversarialNetwork. 2**017**, arXiv:1609.04802v5(25 May).

[17] Dahl R, Norouzi M, Shlens J (2017). Pixel Recursive Super Resolution. arXiv.

[18] Cui, Z., Chang, H., Shan, S., Zhong, B. & Chen, X. Deep network cascade for image super-resolution. *Proc*. *IEEE**Eur*. *Conf*. *Comput*. *Vis*. 1–16 (2014).

[19] Yang, J., Wright, J., Huang, T. & Ma, Y. Image super-resolution as sparse representation of raw image. *IEEE Computer Vision and Pattern Recognition*. 1–8 (2008).

[20] Yang J, Wright J, Huang TS, Ma Y (2010). Image Super-Resolution via Sparse Representation. IEEE Transactions on Image Processing..

[21] Aly HA, Dubois E (2005). Image up-sampling using total-variation regularization with a new observation model. IEEE Transactions on Image Processing.

[22] Zeyde, R., Elad, M. & Protter. M. On single image scale-up using sparse-representations. *Curves and Surfaces* 711–730 (Springer, 2012).

[23] Martin D, Fowlkes C, Tal D, Malik J (2001). A database of human segmented natural images and its application to evaluating segmentation algorithms and measuring ecological statistics. IEEE International Conference on Computer Vision (ICCV).

[24] Abadi M (2016). TensorFlow: Large-scale machine learning on heterogeneous systems. arXiv.

[25] Jinnai H, Spontak RJ (2009). Transmission Electron Microtomography in Polymer Research. Polymer.

[26] Jinnai H, Spontak RJ, Nishi T (2010). Transmission Electron Microtomography and Polymer Nanostructures. Macromolecules.

[27] Loos J (2009). Electron Tomography on Micrometer-Thick Specimens with Nanometer Resolution. Nano Lett..

[28] Loos J, Sourty E, Lu K, de With G (2009). & v. Bavel. S. Imaging Polymer Systems with High-Angle Annular Dark Field Scanning Transmission Electron Microscopy (HAADF−STEM). Macromolecules.

[29] Lu K, Sourty E, Guerra R, Bar G, Loos J (2010). Critical Comparison of Volume Data Obtained by Different Electron Tomography Techniques. Macromolecules.

[30] Jinnai H (2007). Three-Dimensional Structure of a Nanocomposite Material Consisting of Two Kinds of Nanofillers and Rubbery Matrix Studied by Transmission Electron Microtomography. Macromolecules.

[31] Akutagawa K (2008). Mesoscopic Mechanical Analysis of Filled Elastomer with 3D-Finite Element Analysis and Transmission Electron Microtomography. Rubber Chem. Technol..

[32] Yuasa, T., Tominaga, T. & Sone, T. Analysis of Filler Aggregation in Compounds Using Small-angle X-ray Scattering: Effect of Functional Group Introduced into Polymer-ends of Solution-polymerized SBR. *Nippon Gomu Kyokaish*i **8**6, 249–255 (2013), in Japanese. Translation is given in *Int*. *Polym*. *Sci*. *Technol*. **41(2**), T7-T14 (2014).

[33] Baeza GP (2013). Multiscale Filler Structure in Simplified Industrial Nanocomposite Silica/SBR Systems Studied by SAXS and TEM. Macromolecules.

[34] Baeza GP (2013). Effect of Grafting on Rheology and Structure of a Simplified Industrial Nanocomposite Silica/SBR. Macromolecules.

[35] Hagita K, Tominaga T, Sone T (2018). Large-scale reverse Monte Carlo analysis for the morphologies of silica nanoparticles in end-modified rubbers based on ultra-small-angle X-ray scattering data. Polymer.

[36] Vilgis, T. A., Heinrich, G. & Klüppel, M. Reinforcement of Polymer Nano-Composites. (Cambridge, 2009**)**.

[37] Mark, J. E., Erman, B. & Roland, M. The Science and Technology of Rubber, Fourth Edition (Academic Press, 2013**)**.

[38] Uchic MD, Groeber MA, Dimiduk DM, Simmons JP (2006). 3D Microstructural Characterization of Nickel Superalloys via Serial-Sectioning Using a Dual Beam FIB-SEM. Scripta Materialia.

[39] Kato M (2007). Three-dimensional structural analysis of a block copolymer by scanning electron microscopy combined with a focused ion beam. J. Polym. Sci. Part B: Polym. Phys..

[40] OpenCV development team. The OpenCV reference manual. http://opencv.org/ (2015).

[41] Bradski, G. R. & Kaehler, A. Learning OpenCV. (O’Reilly - Sebastopol, CA, 2008).

[42] Kato K (1965). Electron Microscopy of ABS Plastics. J. Electron Microsc..

[43] Kato K (1967). The osmium tetroxide procedure for light and electron microscopy of ABS plastics. Polymer Eng. Sci..

[44] Schneider CA, Rasband WS, Eliceiri KW (2012). NIH Image to ImageJ: 25 years of image analysis. Nature methods.

[45] Thévenaz P, Ruttimann UE, Unser M (1998). A pyramid approach to subpixel registration based on intensity. IEEE Transactions on Image Processing..

